# Factors associated with increased physical activity among patients prescribed physical activity in Swedish routine health care including an offer of counselor support: a 1-year follow-up

**DOI:** 10.1186/s12889-022-12940-4

**Published:** 2022-03-15

**Authors:** Pia Andersen, Sara Holmberg, Kristofer Årestedt, Lena Lendahls, Per Nilsen

**Affiliations:** 1 Department of Research and Development, Region Kronoberg, SE-351 88 Växjö, Sweden; 2 Department of Health, Medicine and Caring Sciences, Division of Society and Health, Linköping University, SE-581 83 Linköping, Sweden; 3 Division of Occupational and Environmental Medicine, Institute of Laboratory Medicine, Lund University, SE-221 00 Lund, Sweden; 4 Faculty of Health and Life Sciences, Department of Medicine and Optometry, Linnaeus University, SE-391 82 Kalmar, Sweden; 5 Faculty of Health and Life Sciences, Department of Health and Caring Sciences, Linnaeus University, SE-391 82 Kalmar, Sweden; 6 The Research Section, Region Kalmar County, SE-391 26 Kalmar, Sweden

**Keywords:** Physical activity on prescription, Counseling, Health care characteristics, Patient characteristics, Predictors

## Abstract

**Background:**

The study addresses knowledge gaps in research regarding influences of routine health care delivery of physical activity on prescription (PAP). The aim was to investigate if patient and health care characteristics are associated with increased physical activity 1 year after prescription among patients offered counselor support in addition to health care professionals’ prescription. The study was conducted in primary and secondary care in a Swedish health care region.

**Methods:**

All PAP recipients during 1 year were invited (*N* = 1503) to participate in this observational prospective study. Data were collected from medical records and questionnaires (baseline and follow-up). Descriptive statistics and multiple logistic regression analysis were used. The outcome variable was increased physical activity after 1 year. Study variables were patient and health care characteristics.

**Results:**

Three hundred and fifty-five patients with complete follow-up data were included. The mean age was 62 years (SD = 14; range, 18–90) and 68% were females. Almost half (47%) had increased physical activity 1 year after PAP. Multiple logistic regression analysis showed that increased physical activity at follow-up was positively associated with lower baseline activity, counselor use, and positive perception of support. Counselor users with low baseline activity had higher odds ratio for increased physical activity at follow-up than non-users (OR = 7.2, 95% CI = 2.2–23.5 vs. OR = 3.2, 95% CI = 1.4–7.5). Positive perception of support was associated with increased physical activity among counselor users but not among non-users.

**Conclusions:**

An increase in physical activity after PAP was related to low baseline activity, positive perception of support, and use of counselor support after PAP. Qualified counseling support linked to PAP seems to be important for achieving increased physical activity among patients with lower baseline activity.

## Background

Physical inactivity is recognized as an important modifiable risk factor for premature death and noncommunicable diseases, posing a major global health threat [1]. There is evidence of the effectiveness of regular physical activity in the prevention and treatment of several diseases, e.g., cardiovascular disease, diabetes, cancer, hypertension, obesity, and depression [2]. However, despite convincing evidence for physical activity, more than a quarter of all adults worldwide do not reach the public health recommendations of at least 150 min of physical activity per week at a moderate to vigorous level, and thereby more than 1.4 billion adults are at risk for development of diseases due to physical inactivity [3].

Health care potentially has an important role in promoting physical activity because many individuals who visit health care for various health problems have a higher probability of being physically inactive [4, 5]. Sweden and several other countries have implemented physical activity on prescription (PAP) programs to achieve increased physical activity among patients consulting health care [6–10]. The PAP program in Sweden is promoted in the Swedish National Board of Health and Welfare’s National Guidelines for Methods of Preventing Disease [11] and by the Swedish Public Health Agency [12]. The program was first implemented in 2001 as part of a national campaign “Sweden on the move” and is now used by all 21 regions responsible for providing health care in Sweden. Recently, the Swedish PAP program was chosen as a best practice program by the European Commission to be implemented in other European Union member states [13, 14].

Despite widespread implementation of PAP in Sweden, there has been some debate whether there is sufficient evidence of its effectiveness to support continued use of the program in routine practice [15–17]. Several studies have generated evidence of the Swedish PAP program in terms of patients’ adherence to the written prescription of physical activity [18], and regarding patients’ changes in physical activity levels [19–22] and other health-related outcomes [19, 21–24]. However, few studies have been conducted under circumstances of routine care to investigate the factors that have influenced changes in physical activity among patients receiving PAP in health care. The existing studies of PAP in Sweden have not provided any information about how the components of the program, such as counseling and support after receiving PAP, influenced changes in physical activity after prescription [18, 25, 26]. Qualitative research from a patient perspective has highlighted that patients need support to be able to change physical activity after receipt of PAP [27, 28]. Studies suggest the importance of social support for patients receiving PAP [29–31]. Previous studies by the researchers behind the present study have indicated that counselor support in association with delivery of PAP is beneficial for increased physical activity [28, 32]. However, the association between counselor support and other factors has not been elucidated.

Addressing key knowledge gaps in research on the Swedish PAP program, the aim of this study was to investigate the patient and health care characteristics associated with increased physical activity 1 year after prescription among patients prescribed physical activity in routine care with access to counselor support. This knowledge is important for improved understanding of the factors that influence patients’ physical activity after PAP [33], especially regarding counselor support. Such knowledge could be important for further development of delivery of PAP in routine health care. This study also adds information on patients receiving PAP in both primary and secondary care, which is important considering that most previous PAP studies in Sweden and other countries focused only on primary care.

## Methods

### Study design

This is a prospective observational study including 1-year follow-up of adult patients prescribed physical activity in routine care, including an offer of counselor support.

### Setting

The study was conducted in one of the 21 decentralized health care regions in Sweden. This southern health care region includes 32 health care centers (primary care), two hospitals for specialized somatic care, and one clinic for specialized psychiatric care (secondary care). Of the 32 health care centers, 11 are privately operated. The Swedish regions are funded by taxes (including privately operated primary care centers); health care is universal for all citizens and patient out-of-pocket fees are low.

The health care region provides care to a total population of approximately 200,000 people. About 80% of the residents are 18 years or older, and about 27% of the population is 65 years or older. This population has somewhat higher life expectancy for both females and males (84.6 years vs. 81.7 years) compared with the rest of Sweden (84.3 years vs. 80.8 years). Furthermore, compared with the national Swedish population, this county has fewer adults with a university education (34% vs. 39%) and more adults with good self-rated health (74% vs. 72%). The proportion of adults who reach at least 150 min of moderate to vigorous physical activity is consistent with that of the general Swedish population (65%). More males than females reach this recommended level of activity (65% versus 61%) [34]. However, in Kronoberg as in the rest of the Swedish regions there are intraregional health differences in the population that need to be considered [35].

### The Swedish PAP program and health care delivery of PAP

The Swedish PAP program involves person-centered prescription of physical activity, counseling, and follow-up [6]. The prescribed activities are intended to be implemented as part the patient’s ordinary life. Prescribed activities can be home-based (e.g., cycling, walking, and Nordic walking) and/or organized (e.g., in fitness centers and sport clubs). When PAP is found to be a suitable intervention for the patient, all licensed health care professionals can prescribe PAP. The FYSS textbook (Physical Activity in Prevention and Treatment of Diseases) can be used as an evidence-based guide by the health care professionals who prescribe PAP [2]. Because there are no guidelines on how the Swedish PAP program should be delivered, health care organizations develop their own mode of delivery of the program [6].

In 2009 and 2010, a structure for routine health care delivery of the Swedish PAP program was implemented in the health care region under study. This mode of delivery is still in use and includes an offer of support from a physical activity counselor to all patients in addition to the health care professional’s PAP. The counselors are licensed health care professionals, typically nurses and physiotherapists, who are employed with the task of supporting patients prescribed physical activity. Their work covers the entire region and their organizational belonging is in a PAP counselor unit within the primary health care setting. Patients are able to meet with a counselor in the PAP counselor unit or at a place that the patient might find more accessible e.g. at their primary care center. The counselors are trained in motivational interviewing (MI) and are familiar with activities offered by local sport clubs and fitness centers. The purpose of including physical activity counselors was to provide patients with support to change their participation in physical activity and to support the health care professional’s delivery of PAP.

All patients receiving PAP in primary and secondary care have access to physical activity counselor support in the year after prescription or longer, if needed. Written information about how to contact a counselor is given by the prescriber of PAP and is automatically printed along with the electronic prescription of physical activity. The patients who accept the offer of support contact a counselor and schedule time and location for the visit. The scheduled time for the first visit is 60 min, while follow-up visits can be up to 30 min. At the first visit the counselor initiates a broader lifestyle dialogue to provide a context for physical activity discussions. There are no patient fees associated with counselor visits, and there are no pre-determined number of visits. The mean number counselor visits by the patients in the present study who used this support was 4.2 (SD = 2.1), with the number of visits ranging from 1 to 11. Any fees associated with the activities are paid by the patient.

### Study population

Patients 18 years or older who received PAP in primary and secondary care between June 2013 and June 2014 were identified through the electronic medical record system. Postal invitations to participate in this study were sent to all identified patients 2 to 3 weeks after the PAP prescription date. One reminder was sent to non-responders after 2 to 3 weeks later. Those who responded to the baseline questionnaire enclosed in the invitation agreed to participate. The baseline responders were sent a follow-up questionnaire after 1 year. The recruitment process has been described previously by the authors of this study [32].

**Fig. 1:**
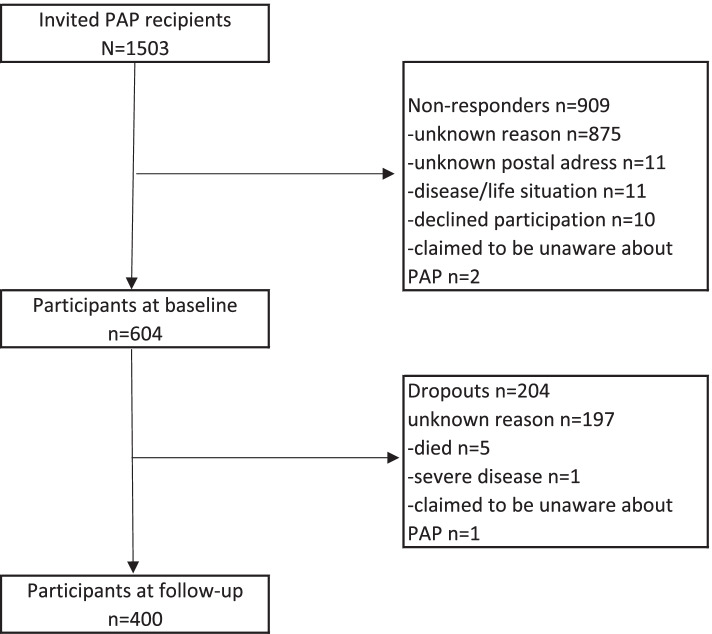
Flow chart of the recruitment process and patient’s participation at baseline and follow-up

A total of 1503 patients were invited, and 604 participated at baseline (Fig. 1). The patients who did not participate were significantly younger (mean age, 50.4 years vs 59.6 years, *p* <  0.001) and more frequently prescribed in secondary care (28% vs 16%, *p* = 0.001) than the participants. No difference was found for sex (*p* = 0.300). Among those invited in secondary care, patients prescribed in psychiatric care were more frequently non-participants (76%) than patients prescribed in somatic care.

Of the 604 participants at baseline, 400 (66%) responded at follow-up (Fig. 1). The 204 dropouts at follow-up were significantly younger (*p* <  0.001) than the patients who participated, they used counselors less frequently (*p* = 0.042), had fewer circulatory diseases (*p* = 0.007), and more mental health disorders (*p* = 0.005). The dropouts did not differ in any of the other variables analyzed (Table 1). Complete data on the patients’ everyday activities and exercise training at baseline and at follow-up was an obligation for analysis in this study. Of the 400 participants at follow-up, 355 (89%) patients were available for this study.

**Table 1:** Patient characteristics at baseline and differences in characteristics between dropouts and participants at follow-up

	Baseline (*n* = 604)	Follow-up		*P* value
		Dropouts (*n* = 204)	Participants (*n* = 400)	
Age (years), mean (SD) [min–max]	59.6 (15.2) [18–90]	54.9 (16.4) [18–86]	62.0 (14.0) [18–90]	< 0.001^a^
Sex, female n (%)	402 (66.6)	126 (61.8)	276 (69.0)	0.075^b^
Education, university education n (%)	145 (24.9)	42 (21.3)	103 (26.7)	0.156^b^
Baseline activity (score 3–19), median (IQR) [min–max]	9.0 (5.0) [3–19]	8.0 (5.0) [3–19]	9.0 (5.0) [3–19]	0.004^c^
Confident to change (1–10), median (IQR) [min–max]	7.0 (3.0) [1–10]	6.5 (3.0) [1–10]	7.0 (3.0) [1–10]	0.593^c^
Important to change (1–10), median (IQR) [min–max]	8.0 (3.0) [1–10]	8.0 (3.0) [1–10]	7.0 (4.0) [1–10]	0.266^c^
Diagnoses^d^				
Musculoskeletal diseases, yes n (%)	341 (58.9)	102 (54.3)	239 (61.1)	0.116^b^
Circulatory diseases, yes n (%)	284 (49.1)	77 (41.0)	207 (52.9)	0.007^b^
Endocrine diseases, yes n (%)	222 (38.3)	66 (35.1)	156 (39.9)	0.267^b^
Mental health disorders, yes n (%)	171 (29.5)	70 (37.2)	101 (25.8)	0.005^b^
Respiratory diseases, yes n (%)	168 (29.0)	64 (34.0)	104 (26.6)	0.065^b^
Number of diagnoses, median (IQR) [min–max]	7.0 (5.00) [0–18]	6.00 (5.00) [0–15]	7.00 (5.00) [0–18]	0.887^c^
Health care use^e^				
Number of office visits, median (IQR) [min–max]	13.0 (21.0) [1–119]	13.0 (22.0) [1–116]	14.0 (19.0) [1–119]	0.715^c^
≥ 1 hospital stay, yes n (%)	148 (24.5)	49 (26.1)	99 (25.2)	0.821^b^
Prescribing professional group, physician n (%)	169 (28.0)	58 (28.4)	111 (27.8)	0.860^b^
Prescribing setting, primary care n (%)	490 (81.1)	158 (77.5)	332 (83.0)	0.099^b^
Used counselor support after PAP, yes n (%)	208 (34.4)	59 (28.9)	149 (37.3)	0.042^b^

^a^Independent sample t test

^b^Pearson chi-squared test (binary variables)

^c^Mann-Whitney U test

^d^Medical record registrations 12 months before PAP of the International Classification of Diseases version 10 (ICD 10) classified diagnostic groups excluded Z

^e^Medical record registrations 12 months before PAP of health care visits in primary and/or specialized somatic and psychiatric care with no selection of professional, overnight hospital stays, or specialized somatic and psychiatric care

### Data collection and measurements

Data were collected from electronic medical records and a self-administrated questionnaire at baseline and at follow-up.

### Outcome of physical activity

The outcome variable was increased physical activity between baseline and follow-up. Physical activity data were collected by questionnaire and measured by one question on everyday activity and one question on exercise training at baseline and at follow-up. The patients were asked: “During a regular week, how much time are you physically active in ways that are not exercise, for example walks, bicycling, or gardening? Add together all activities lasting at least 10 min”; and “During a regular week, how much time do you spend exercising on a level that makes you short of breath, for example, running, fitness class, or ball games?” [36]. These questions have been validated and are recommended for use in clinical practice by the Swedish National Board of Health and Welfare [36, 37]. The responses to these questions are calculated in a scale score of weekly physical activity ranging from 3 (no activity minutes) to 19 (> 300 min of everyday activity and > 120 min of exercise training per week). The cutoff value for reaching the recommended level of at least 150 min of physical activity per week is 11. The score of 11 (±1) was used as the cutoff for categorization of patients’ physical activity at baseline into three categories: 3 to 9 was categorized as < 150 activity minutes, 10 to 12 as ~ 150 activity minutes, and 13 to 19 as > 150 activity minutes. These categories were used as explanatory variables in the multiple logistic regression model.

### Patient characteristics

Data on the patients’ age, sex, diagnoses, and health care consumption were collected electronically through the medical record system. Age was measured at PAP date. Sex was recorded as female and male. Details of all registered diagnoses, office visits to health care, and occasions of inpatient care were collected in the 12 months before the prescription date. Diagnoses were described at a disease group level, i.e. by the first character code according to the International Classification of Diseases version 10 (ICD-10) [38]. The five most common disease groups were identified; i.e., musculoskeletal diseases, circulatory diseases, endocrine diseases, mental health disorders, and respiratory diseases. Each of these disease groups were dichotomized as “yes” or “no” according to their presence within the disease group. The number of disease groups and number of health care office visits was calculated for each patient. Inpatient care was dichotomized as “yes” or “no” according to whether one or more occasion of inpatient care occurred. Data on age, sex, and number of disease groups were used as an explanatory variable in the multiple logistic regression model.

Data on the patients’ education level were collected in the baseline questionnaire and assessed in terms of the patient’s highest education level attained and dichotomized into having a university degree, “yes” or “no”. Patients were asked about their highest education level in a four-item question, and university education was one of the options. Education level was used as an explanatory variable in the multiple logistic regression model.

Data on the patients’ readiness to change was collected through the baseline questionnaire and measured by two questions derived from MI and recommended for clinical practice of PAP [2]. Patients were asked “how important is it for you to change your physical activity” and “how confident are you about succeeding in changing your physical activity”. The responses options were on a Likert scale ranging from 1 to 10, where 1 was equal to “not at all …” and 10 to “very …”. These measurements were used in the description of the patient characteristics.

Data on exercise among family and friends were collected through the baseline questionnaire and measured by the question “How many of your friends, family exercise regularly?” The four alternatives were “none”, “some”, “a majority” and “all”. The answers were categorized as “yes” and “no”, where yes included “a majority” and “all” and no the other two alternatives. This question has previously been used in a Swedish survey for adults aged 20 to 65 years [39]. Exercise among family and friends was used as an explanatory variable in the multiple logistic regression model.

Patients’ perception of received support after PAP was collected by the follow-up questionnaire and measured by the question “Do you feel that you have received the support you needed to increase your physical activity” Three alternatives for response was given: “yes”, “no” and “do not remember”. The answers were categorized into received needed support for increased physical activity, “yes” or “no”. “No” encompassed both “no” and “do not remember”. This question was developed by the researchers of the study with the purpose to complement the measurements of patients’ use of counselor support. Patients’ perception of support was used as an explanatory variable in the multiple logistic regression model.

### Health care characteristics

Health care characteristics included data on the prescribing professional and the setting, and the patient’s counselor visits. These data were collected electronically through the medical record system. The prescribing professional was categorized as physician: “yes” or “no”. “No” included prescription from others, i.e., physiotherapists, nurses, psychologists, behavioral therapists, midwifes, dieticians, and occupational therapists. The prescribing setting was categorized “yes” or “no” for PAP in primary care. “No” included prescription in secondary care (somatic and psychiatric). All data concerning face-to-face consultation with a counselor were collected in the first 16 months after PAP and were dichotomized as “yes” or “no” for use of counselor support. “Yes” included patients with one or more face-to-face consultations with a counselor. All measurements of health care characteristics were used as explanatory variables in the multiple logistic regression model.

### Statistical analysis

Descriptive statistics were used for presentation of the patient characteristics and study variables. Continuous data are presented as means, standard deviation (SD), and min–max. Non-normally distributed continuous data and ordered categories are presented as medians, interquartile range (IQR), and min–max, while non-ordered categorical data are presented as frequencies. Depending on the data level and distribution, chi-squared statistics, Mann-Whitney U test, or independent sample t test was used to compare groups.

Multiple binary logistic regression analysis (forced entry) was used to determine the relationship between the explanatory variables and the outcome variable. The outcome variable “increased physical activity” was calculated by subtract physical activity at the follow-up from physical activity at baseline on an individual level. The difference score was then recoded so that 0 reflected no change or decreased physical activity (i.e., difference scores ≤0) while 1 reflected increased psychical activity (i.e., difference score > 0).

The explanatory variables included age (continuous), sex (female = 1, male = 0), university education (yes = 1, no = 0), baseline activity (score 3–9 = 1, score 10–12 = 2, score 13–19 = REF), number of diagnostic groups (continuous), exercise among family and friends (yes = 1, no = 0), prescription by physician (yes = 1, no = 0), prescription in primary care (yes = 1, no = 0), use of counselor support (yes = 1, no = 0), and positive perception of support for increased physical activity after PAP (yes = 1, no = 0).

All explanatory variables were included in a multiple binary logistic regression model, including an analysis of the whole sample and of two stratified subgroups. One subgroup included the patients who used counselor support and one group included the patients who did not use this support.

A *p* value < 0.05 was regarded as statistically significant. All statistical analyses were performed with SPSS Statistics for Windows 27.0 (IBM Corp, Armonk, NY, USA).

## Results

### Study sample

The mean age of the study sample (*n* = 355) was 61.1 (SD = 14.2). More than half were females (*n* = 242, 68%) and about one quarter had a university education (*n* = 92, 27%) (Table 2). Low physical activity (score 3–9) at baseline was seen in half of the study sample. Several of the patients had a high number of diagnoses and a high number of health care visits before PAP. Musculoskeletal disease was the most common disease (*n* = 254, 59%), followed by circulatory disease (*n* = 179, 52%), endocrine disease (*n* = 135, 38%), respiratory disease (*n* = 90, 26%), and mental health disorders (*n* = 89, 26%). PAP was most often prescribed in primary care (*n* = 298, 84%) and by other health care professionals than physicians (*n* = 257, 72% vs. 98, 28%), and the offer of counselor support was used by less than two-fifths of the patients (n = 135, 38%).

**Table 2:** Characteristics of all patients in the study and in patients with increased physical activity versus patients with no increase in physical activity

	Study sample (*N* = 355)	Increased physical activity		*P* value	Valid data
		Yes (*n* = 167)	No (*n* = 188)		
Age (years), mean (SD) [min–max]	61.6 (14.2) [18–90]	59.6 (13.8) [18–85]	63.3 (14.3) [23–90]	0.013^a^	355
Sex, female n (%)	242 (68.2)	117 (70.1)	125 (66.5)	0.471^b^	355
Education, university education n (%)	92 (26.6)	49 (29.9)	43 (23.6)	0.189^b^	346
Baseline activity (score 3–19), median (IQR) [min–max]	10.0 (5.0) [3–19]	8.0 (5.0) [3–18]	11.0 (4.8) [3–19]	< 0.001^c^	355
< 150 min (score 3–9)	176 (49.6)	105 (62.9)	71 (37.8)		
~ 150 min (score 10–12)	95 (26.8)	39 (23.4)	56 (29.8)		
> 150 min (score 13–19)	84 (23.7)	23 (13.8)	61 (32.4)		
Confidence to change (1–10), median (IQR) [min–max]	7.0 (3.0) [1–10]	7.0 (3.0) [1–10]	7.0 (3.0) [1–10]	0.643^c^	351
Important to change (1–10), median (IQR) [min–max]	8.0 (4.0) [1–10]	8.0 (3.0) [1–10]	8.0 (4.0) [1–10]	0.152^c^	353
Exercising family/friends, yes n (%)	151 (43.3)	58 (35.4)	93 (50.3)	0.005^b^	349
Diagnoses^d^					
Musculoskeletal diseases, yes n (%)	204 (59.0)	87 (54.0)	117 (63.2)	0.082^b^	346
Circulatory diseases, yes n (%)	179 (51.7)	78 (48.4)	101 (54.6)	0.254^b^	346
Endocrine diseases, yes n (%)	135 (38.4)	57 (35.4)	76 (41.1)	0.279^b^	346
Mental health disorders, yes n (%)	89 (25.7)	48 (29.8)	41 (22.2)	0.104^b^	346
Respiratory diseases, yes n (%)	90 (26.0)	42 (26.1)	48 (25.9)	0.976^b^	346
Number of diagnoses, median (IQR) [min–max]	6.0 (5.0) [0–18]	6.0 (4.0) [0–16]	7.0 (4.5) [0–18]	0.006^c^	346
Health care use^e^					
Number of office visits, median (IQR) [min–max]	12.0 (19.0) [1–118]	11.0 (18.0) [1–92]	15.0 (15.0) [1–118]	0.038^c^	348
≥ 1 hospital stay, yes n (%)	86 (24.1)	38 (28.3)	48 (25.9)	0.570^b^	348
Prescribing professional group, physician n (%)	98 (27.7)	136 (81.4)	162 (86.2)	0.225^b^	355
Prescribing setting, primary care n (%)	298 (84.0)	52 (31.1)	46 (24.5)	0.161^b^	355
Used counselor support after PAP, yes n (%)	135 (38.0)	82 (49.7)	52 (27.7)	< 0.001^b^	355
Positive perception of received support, yes n (%)	211 (60.3)	115 (69.3)	96 (52.2)	< 0.001^b^	350

^a^Independent sample t test

^b^Pearson chi-squared test (binary variables)

^c^Mann-Whitney U test

^d^Medical record registrations 12 months before PAP of the International Classification of Diseases version 10 (ICD 10) classified diagnostic groups excluded Z

^e^Medical record registrations 12 months before PAP of health care visits in primary and/or specialized somatic and psychiatric care with no selection of professional, overnight hospital stays, or specialized somatic and psychiatric care

### Differences in characteristics between patients with increased activity versus patients with no increase in activity

Almost half of the patients (*n* = 167, 47%) reported increased activity 1 year after PAP. In contrast, 59 (17%) patients reported unchanged physical activity and 129 (36%) reported decreased physical activity.

The patients with increased activity were somewhat younger (*p* = 0.013), had a lower level of baseline activity (*p* < 0.001), had fewer exercising family and friends (*p* = 0.005), and had fewer health care visits (*p* = 0.038) and diagnoses (*p* = 0.009) than patients with no increase (Table 2). A higher proportion of those who used counselors (*p* < 0.001) and patients with a positive perception of support after PAP (*p* < 0.001) were seen among patients with increased activity.

The baseline median score for patient’s confidence to change and for patient’s perception of the importance of changing was high and did not differ between the patients with increased activity and the patients with no increase in activity 1 year after PAP (Table 2).

### Multiple binary logistic regression analysis

The multiple binary logistic regression model of the whole sample showed that lower levels of baseline activity, use of counselor support, and having a positive perception of support received after PAP were significantly and positively associated with increased physical activity 1 year after PAP (Table 3).

**Table 3:** Multiple binary logistic regression analysis of factors associated with improved physical activity 1 year after prescription

	Whole sample (*n* = 326)^a^		Counselor yes (*n* = 125)		Counselor no (*n* = 201)	
	Odds ratio (95% CI)	*p* value	Odds ratio (95% CI)	*p* value	Odds ratio (95% CI)	*p* value
Age	0.99 (0.97–1.00)	0.189	0.99 (0.96–1.02)	0.450	0.99 (0.97–1.01)	0.344
Female	1.12 (0.66–1.92)	0.668	1.50 (0.59–3,81)	0.392	0.99 (0.50–1.96)	0.970
University education	1.30 (0.75–2.27)	0.346	1.35 (0.54–3.41)	0.522	1.07 (0.51–2.23)	0.865
Baseline activity						
< 150 min (score 3–9)	4.33 (2.24–8.38)	< 0.001	7.17 (2.19–23.48)	0.001	3.23 (1.40–7.47)	0.006
~ 150 min (score 10–12)	2.33 (1.14–4.78)	0.021	4.22 (1.10–16.21)	0.036	1.80 (0.74–4.34)	0.194
> 150 min (score 13–19)	Reference		Reference		Reference	
Number of diagnoses^b^	0.93 (0.87–1.00)	0.064	0.89 (0.79–1.01)	0.081	0.95 (0.87–1.05)	0.301
Exercising friends/family	0.66 (0.40–1.08)	0.100	0.77 (0.40–1.89)	0.579	0.60 (0.32–1.13)	0.112
Prescribed by physician	1.15 (0.66–1.98)	0.626	0.96 (0.37–2.50)	0.937	1.27 (0.63–2.59)	0.514
Prescribed in primary care	0.78 (0.40–1.60)	0.465	1.38 (0.42–4.56)	0.594	0.65 (0.28–1.52)	0.320
Counselor users	1.80 (1.07–3.00)	0.026	Strata		Strata	
Positive perception of support	2.08 (1.24–3.48)	0.005	7.24 (2.64–19.85)	< 0.001	1.22 (0.65–2.27)	0.537
Pseudo R square: Cox & Snell/Nagelkerke	0.170/0.227		0.238/0.323		0.103/0.140	

^a^Comprise the 326 patients with complete data for all variables in this analysis

^b^Medical record registrations 12 months before PAP of the International Classification of Diseases version 10 (ICD 10) classified diagnostic groups excluded Z

The multiple binary logistic regression model, in which patients were stratified according to whether they had used or not used counselor support after PAP showed different results for the groups. Among counselor users, increased physical activity was found to be associated with lower levels of baseline activity and having a positive perception of support given after PAP. The other factors were not associated with increased physical activity: age, gender, education, number of diagnoses, having exercising friends or family, being prescribed by a physician and being prescribed in primary care. Among non-counselor users, increased activity was only associated with low baseline activity (Table 3). Among the counselor users, 99 patients (74.4%) reported a positive perception of support, and the corresponding figure among the non-counselor users was 112 (51.6%).

## Discussion

This study sought to investigate patient and health care characteristics associated with increased physical activity 1 year after prescription among patients prescribed physical activity in routine care. We found that increased physical activity was associated with having a baseline activity below the recommended level of at least 150 min of physical activity per week, using counselor support, and having a positive perception of support received after PAP.

The positive association found in this study between lower baseline activity and increased physical activity after 1 year indicates that PAP was an appropriate intervention for patients with the greatest need for increased activity. Similar results were seen in a previous Swedish PAP study including 6 months follow-up [26]. Contrary to our findings, higher baseline activity was found in others studies to be associated with higher adherence to the written prescription of physical activity [18] and patients’ adherence to referred exercise programs [40]. However, these inconsistent findings might be due to differences in measuring adherence and increased activity. Compared with other studies of PAP [18, 21, 22, 41], several patients in our study had a relatively high level of activity when they received PAP.

There are a few potential explanations for the high level of baseline activity. Since the baseline measure of physical activity was included in the questionnaire, which was sent to the patients after they received PAP, some patients might already have increased their physical activity when they responded to the questionnaire. Other explanations might be that the present study reached more patients with higher activity levels or the fact that PAP was not only prescribed to patients with insufficient physical activity. The patients’ physical activity might have been poorly assessed by the health care professionals at the time of the prescription [42]. Available tools for measurement have been shown to be impractical in clinical work [43]. Health care professionals may have relied on their own opinions about physical activity rather than using recommended physical activity guidelines [42, 44]. For this study, we have no information on how the patients’ physical activity was assessed by individual prescribers of PAP. However, lack of registration of patients’ physical activity in the medical records indicates that assessment of physical activity was not frequently carried out. Improved attention to assessment of patients’ physical activity in health care practice before initiating a PAP could yield improved results.

The patients’ positive perception of support in this study is likely related to the use of counselor support although the questionnaire did not specifically ask what this support entailed. A qualitative interview study which included PAP recipients recruited from the same population as the present study showed that patients who visited a counselor expressed a great deal of confidence in the counselor’s competence and in the individualized support given by the counselor [28]. The need for support when receiving PAP has been reported in several qualitative studies [27–29, 31]. Support from health care professionals seems to be particularly important among patients with disease [27, 45]. It has been documented that some patients are afraid that physical activity might worsen the disease [46].

Our finding was somewhat unexpected regarding the negative association between patients’ increase in physical activity and the presence of family and friends who exercise. Social influence is a key element in shaping attitudes, beliefs, and behaviors in numerous social cognitive theories, e.g., Theory of Planned Behavior [47], Social Cognitive Theory [48] and Self-Determination Theory [49]. However, our result may be explained with reference to Social Comparison Theory [50], which posits that people have an innate drive to evaluate themselves in comparison with others. Thus, in comparison with others performing better (upward comparisons), there is a risk of underestimation of one’s own ability. In contrast, we can devote ourselves to comparison with others performing worse (downward comparison) than ourselves, because it makes us feel more satisfied with ourselves [51]. Among PAP recipients, comparisons with peers in group activities at similar or lower capability to perform activity has been reported to strengthen satisfaction with one’s own capability [28, 31]. It is not clear whether a different question than asking about exercising family and friends would be more informative or if support should be measured at follow-up. The results in our study regarding patients’ perception of support indicate that follow-up measures of support might better predict patients increase in physical activity. Results in a Swedish PAP study that used a validated instrument to measure support from family and friends found that neither family nor friends was a significant baseline predictor for PAP recipients’ increase in physical activity [26]. However, the adjusted analysis in the present study showed no significant association with increased physical activity for friends and family who exercise. Measurement of support by family and friends seems to be complex issue that needs more attention in research.

In our study, the adjusted analysis showed no significant association with increased activity for age, sex, education level, or number of diagnoses. Studies have shown different findings regarding the influence of patients’ age [18, 26, 52] and sex [18, 26, 53]. These differences might be influenced by the use of different outcome measures and variations concerning the health care delivery of PAP [54]. For example, older age has been found to be positively associated with adherence to prescribed exercise programs [52] and the written physical activity prescription [18] but not to increased physical activity after PAP [26]. It is well established that the behavior change involved in increased physical activity is complex. Thus, several factors influence the changes, including not only patients’ physical and psychological capacity, attitudes and motivation, but also their inner context (e.g., family and friends) and outer context (e.g., access to local sports activities and structural and economic constraints of health care) [33].

There are some limitations that need to be considered when interpreting the results of the present study. The study was conducted over 1 year, which makes it impossible to assess how the physical activity levels developed in a longer term. We recruited 40% of all PAP recipients at baseline within primary and secondary routine care over the 1-year period. Two-thirds of the baseline participants participated at follow-up. Patient-related willingness to participate is difficult to overcome in the type of real-world studies that we undertook. Non-participants at both baseline and follow-up were younger and had more mental health disorders diagnoses.

Due to lack of registration of patients’ physical activity in the medical records, it was not possible to collect these data on the day when PAP was prescribed. Measures of patients’ physical activity were based on responses from the baseline questionnaire, which were sent to the patients 2 to 3 weeks after the prescription day. The patients who did not respond were sent a reminder after an additional 3 weeks. However, if patients responded directly or after a reminder did not influence the outcome of physical activity at follow-up (*p* = 0.101). The use of self-reported measurement of physical activity could be seen as a limitation of the study.

From the medical records, it was not possible to retrieve any data on support from health care professionals other than the physical activity counselors. Other health care professionals might have provided support after PAP. Support might also have been given from others outside health care.

The limitations of the study should be balanced against the study’s strengths. To our knowledge, this is the first routine health care study of the Swedish PAP program investigating the association between counselor support after PAP and increase in patients’ physical activity. Real-world health care studies including routine health care populations are essential to document the benefits of interventions as delivered in routine clinical practice. This type of study typically has high generalizability, clinical utility and policy relevance [55]. The study reflected real-world PAP delivery, had a relatively large sample of PAP recipients recruited, had high data quality with low internal non-response, and had a 1-year recruitment period, which reduced the risk of bias related to seasonal variations of physical activity [56]. Recruitment of participants and data collection in this study were handled by one of the researchers, therefore routine health care delivery of PAP by health care professionals and counselors was not influenced by research-related tasks.

### Implications for clinical practice

Identifying patients with insufficient levels of activity might increase the quality of routine health care delivery of PAP, and allow health care counseling resources to be allocated to patients with the greatest need for increased activity. This study suggests a feasible method for improving the future health and well-being of patients and the results could contribute to health practitioners’ confidence in the outcomes of PAP.

## Conclusion

Increased physical activity after PAP was related to low baseline activity, a positive perception of support, and use of counselor support after PAP. Qualified counseling support linked to PAP seems to be most important for achieving increased physical activity among patients with lower baseline activity.

## Data Availability

The data that support the findings of this study are available from Region Kronoberg, but restrictions apply to the availability of these data, which were used under license for the current study, and so are not publicly available. However, data are available from the corresponding author (PA) upon reasonable request and with permission of Region Kronoberg.

## Abbreviations

ICD 10: International Classification of Diseases version 10
PAP: Physical activity on prescription; used as an overall term for prescribed physical activity in health care
